# COVID-19 and Acute Kidney Injury: A Systematic Review

**DOI:** 10.3389/fmed.2022.705908

**Published:** 2022-04-04

**Authors:** Tahereh Sabaghian, Amir Behnam Kharazmi, Ali Ansari, Fatemeh Omidi, Seyyedeh Neda Kazemi, Bahareh Hajikhani, Roya Vaziri-Harami, Ardeshir Tajbakhsh, Sajjad Omidi, Sara Haddadi, Amir Hashem Shahidi Bonjar, Mohammad Javad Nasiri, Mehdi Mirsaeidi

**Affiliations:** ^1^Clinical Research Development Center, Imam Hossein Educational Hospital, Shahid Beheshti University of Medical Sciences, Tehran, Iran; ^2^Department of Internal Medicine, Imam Hossein Medical Center, School of Medicine, Shahid Beheshti University of Medical Sciences, Tehran, Iran; ^3^School of Medicine, Shahid Beheshti University of Medical Sciences, Tehran, Iran; ^4^Department of Cardiology, Imam Hossein Hospital, Shahid Beheshti University of Medical Sciences, Tehran, Iran; ^5^Department of Obstetrics and Gynecology and Female Infertility Unit, Tehran University of Medical Sciences, Tehran, Iran; ^6^Department of Microbiology, School of Medicine, Shahid Beheshti University of Medical Sciences, Tehran, Iran; ^7^Imam Hossein Hospital, Behavioral Science Research Center of Shahid Beheshti University of Medical Sciences, Tehran, Iran; ^8^Anesthesia Research Centre, Shahid Beheshti University of Medical Sciences, Tehran, Iran; ^9^Department of Pulmonary and Critical Care, University of Miami Miller School of Medicine, Miami, FL, United States; ^10^Clinician Scientist of Dental Materials and Restorative Dentistry, School of Dentistry, Shahid Beheshti University of Medical Sciences, Tehran, Iran; ^11^Division of Pulmonary and Critical Care, College of Medicine-Jacksonville, University of Florida, Jacksonville, FL, United States

**Keywords:** acute kidney injury, COVID-19, SARS-CoV-2, systematic review, AKI

## Abstract

**Introduction:**

Acute kidney injury (AKI) has been associated with an increased mortality rate among hospitalized patients with Coronavirus disease 2019 (COVID-19). The current review aimed to evaluate the symptoms, complications, and treatments performed to manage AKI in patients with COVID-19.

**Methods:**

We searched PubMed/Medline, Web of Science, and Embase for the relevant scientific literature published up to February 1, 2022. The following keywords were used: “COVID-19”, “SARS-CoV-2”, and “Acute kidney injury”.

**Results:**

Forty-four studies with a total number of 114 COVID-19 patients with AKI (Mean age: 53.6 years) were included in our systematic review. The most common comorbidities in patients with COVID-19 suffering from AKI were the history of diabetes, hypertension, and hyperlipidemia. Twelve out of the 44 included studies reported a history of chronic kidney disease (CKD) in this group of patients. Focal segmental glomerulosclerosis (FSGS) and acute tubular necrosis (ATN) were the most common pathological evidence. The average length of hospital stay was 19 days, and the average duration of need for mechanical ventilation was 3 days.

**Conclusions:**

The current systematic review shows that AKI frequently complicates the course of COVID-19 hospitalizations and is associated with increased severity of illness, prolonged duration of hospitalization, and poor prognosis. Given the extent of the adverse impact of AKI, early detection of comorbidities and renal complications is essential to improve the outcomes of COVID-19 patients.

## Introduction

Acute kidney injury (AKI) has been associated with an increased mortality rate among hospitalized patients with Coronavirus disease 2019 (COVID-19). An incidence rate of around 10% was reported in these patients. This incidence could be associated with age, disease severity, and ethnicity. Studies have shown that AKI could be closely related to the effect of severe acute respiratory syndrome coronavirus 2 (SARS-CoV-2), which is the causative agent of COVID-19, on the kidney rather than any side effect of experimental drugs for COVID-19 such as remdesivir (1).

According to a recent meta-analysis incidence of AKI in COVID-19 was 8.9% (2). This was close to the incidence rate of AKI in patients with community-acquired pneumonia. However, there was statistical heterogeneity among the studies (2). Other meta-analysis studies have shown that males have higher mortality among COVID-19 patients (3). Another systematic review and meta-analysis revealed that being male and diabetic in COVID-19 patients were associated with developing AKI (4). Studies from the USA and Europe presented a pooled incidence of 28.6% and 7.7% for AKI, respectively (5). AKI has also been detected as a predictor of fatality and severe COVID-19 infection (5).

Due to the importance of this issue, the current study aimed to evaluate the symptoms, complications, and treatments performed to manage AKI in patients with COVID-19 as a comprehensive systematic review.

## Methods

This review conforms to the “Preferred Reporting Items for Systematic Reviews and Meta-Analyses” (PRISMA) statement (6).

### Literature Search

We searched PubMed/Medline, Web of Science, and Embase for relevant studies, published up to February 1, 2022.

The following terms were used in the search strategy: COVID-19, severe acute respiratory syndrome coronavirus 2, SARS-CoV-2, and acute kidney injury. Only studies written in English were selected.

### Study Selection

The records found through database searching were merged, and the duplicates were removed using EndNote X7 (Thomson Reuters, Toronto, ON, Canada). Two reviewers (TS and FO) independently screened the records by title/abstract and full text to exclude those unrelated to the study objectives. Included studies met the following criteria: (1) COVID-19 patients diagnosed with reference standard test; (2) AKI defined according to the Kidney Disease Improving Global Outcomes (KDIGO) guidelines (7). The KDIGO guidelines define AKI as follows: increase in serum creatinine by ≥0.3 mg/dL (≥26.5 micromol/L) within 48 hours, or increase in serum creatinine to ≥1.5 times baseline, which is known or presumed to have occurred within the prior seven days, or Urine volume <0.5 mL/kg/h for 6 hours. Furthermore, different stages of AKI was defined as follows: Stage 1; increase in serum creatinine to 1.5–1.9 times baseline, or increase in serum creatinine by ≥0.3 mg/dL (≥26.5 micromol/L), or reduction in urine output to <0.5 mL/kg/h for 6–12 h. Stage 2; increase serum creatinine to 2.0–2.9 times baseline, or reduce urine output to <0.5 mL/kg/h for ≥12 hours. Stage 3; increase in serum creatinine to 3.0 times baseline, or increase in serum creatinine to ≥4.0 mg/dL (≥353.6 micromol/L), or reduction in urine output to <0.3 mL/kg/h for ≥24 h, or anuria for ≥12 h, or the initiation of kidney replacement therapy, or, in patients <18 years, decrease in estimated glomerular filtration rate (eGFR) to <35 mL/min/1.73 m^2^.

### Data Extraction

Two reviewers (TS and FO) designed a data extraction form and extracted data from all eligible studies, with differences being resolved by consensus. Data such as country of origin, the number of patients with AKI, the number of patients with confirmed COVID-19, clinical symptoms, laboratory findings, outcomes, diagnostic methods, and treatment were extracted from the selected articles.

## Results

**Figure 1:**
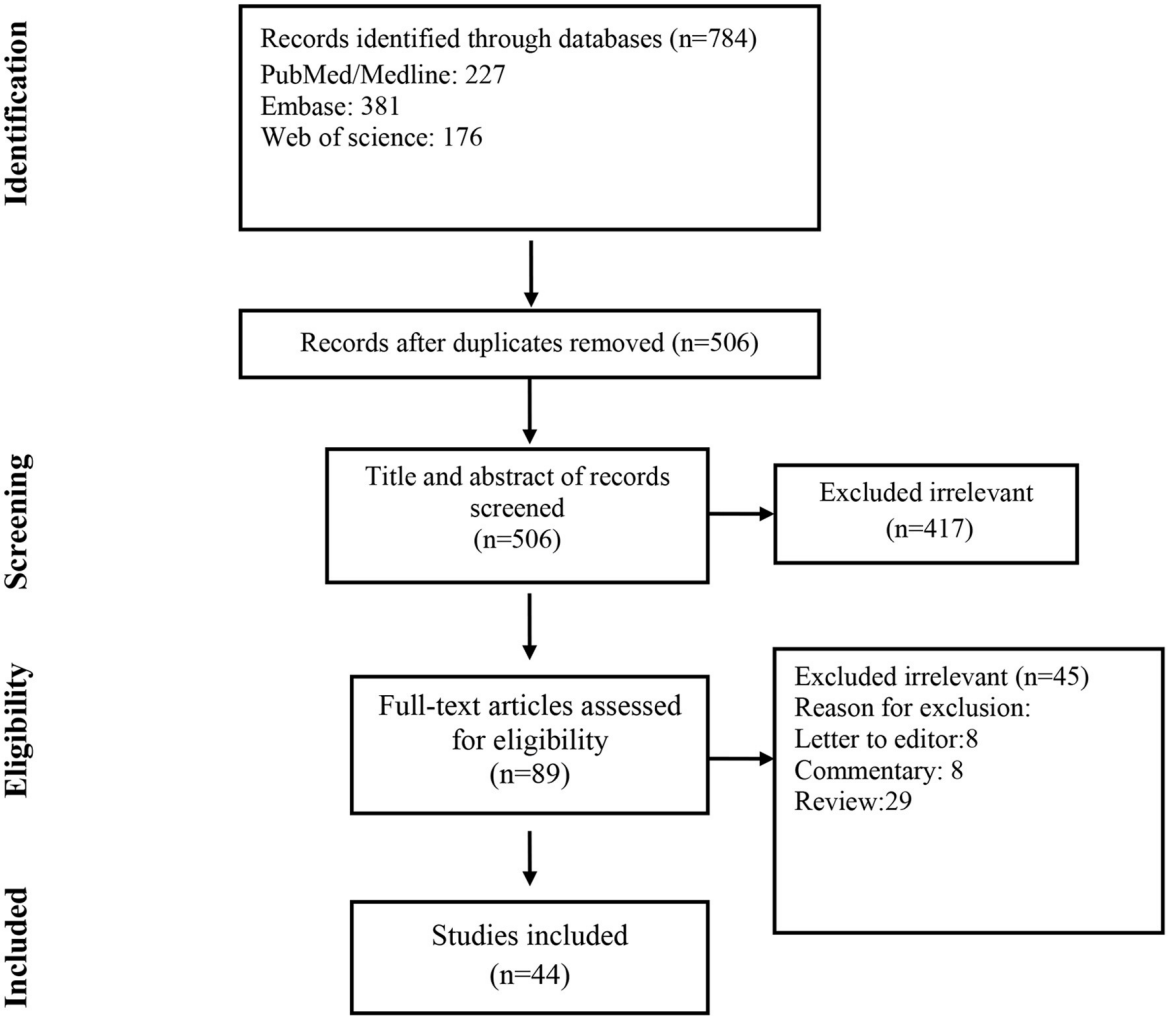
Flow chart of study selection for inclusion in the systematic review.

A total of 784 records were found in the initial search; after removing duplicate articles, the titles and abstracts of 506 references were screened (Figure 1). Of these, 89 articles were selected for a full-text review. After the full-text review, 44 articles met the inclusion criteria.

The KDIGO criteria were used in all selected papers to define AKI. A total of 114 COVID-19 patients with AKI (Mean age: 53.6 years) were included in the current study (Table 1).

**Table 1:** Characteristics of Case reports/case series studies.

**Male/Female**	**Mean age**	**No. of patients with**	**Type of**	**Published**	**Country**	**References**
		**Covid-19 and AKI**	**study**	**time**		
2M/2F	60	4	Case series	2020	USA	Chenna et al. (8)
1F	66	1	Case report	2020	Canada	Chibane et al. (9)
1M	32	1	Case report	2020	Iran	Ghobadi et al. (10)
1M	49	1	Case report	2020	USA	Gopalakrishnan et al. (11)
2M/1F	43	3	Case series	2020	USA	Laurence et al. (12)
1M	62	1	Case report	2020	Greece	Marinaki et al. (13)
1M	46	1	Case report	2020	Mexico	Solís et al. (14)
1M/1F	61.5	2	Case series	2020	China	Wang et al. (15)
1M	67	1	Case report	2020	USA	Padalaa et al. (16)
1M	38	1	Case report	2020	USA	Husain et al. (17)
1M/1F	43	2	Case report	2020	USA	Magoon et al. (18)
1M	49	1	Case report	2020	Italy	Rossi et al. (19)
2M/1F	69.33	3	Case report	2020	USA	Roomi et al. (20)
1F	68	1	Case report	2020	USA	Sise et al. (21)
1F	33	1	Case report	2020	Iran	Taghizadieh et al. (22)
1M	16	1	Case report	2020	USA	Samies et al. (23)
1M	62	1	Case report	2020	Japan	Nagatomo et al. (24)
1M	46	1	Case report	2020	Columbia	Peleg et al. (25)
1M	53	1	Case report	2020	France	Couturier et al. (26)
1M	54	1	Case report	2020	Iran	Vardanjania et al. (27)
1M/1F	58	2	Case report	2020	USA	Sharma et al. (28)
2M	62.5	2	Case series	2020	USA	Gupta et al. (29)
2M	60	2	Case report	2020	Netherland	Post et al. (30)
5M/5F	65	10	Case series	2020	USA	Sharma et al. (31)
10M/2F	70	12	Case series	2020	USA	Golmai et al. (32)
16M/6F	66	22	Case series	2020	Netherland	Wilbers et al. (33)
1M	NR	1	Case report	2021	Japan	Daisuke et al. (34)
1F	70	1	Case report	2021	Greece	Aikaterini et al. (35)
1F	63	1	Case report	2021	Morocco	Oussama et al. (36)
1F	53	1	Case report	2021	Romania	Stefan et al. (37)
7M/4F	50	11	Case series	2021	USA	Singh et al. (38)
1M	68	1	Case report	2021	Netherland	Melchers et al. (39)
1M	62	1	Case report	2021	Switzerland	Szajek et al. (40)
4M/1F	63	5	Case series	2021	China	Chen et al. (41)
1M	71	1	Case report	2020	USA	Melegari et al. (42)
1M	50	1	Case report	2021	USA	Egoryan et al. (43)
1M	62	1	Case report	2020	USA	Ammous et al. (44)
1M	25	1	Case report	2020	Italy	Lenti et al. (45)
1F	66	1	Case report	2020	Canada	Chibane et al. (9)
1F	22	1	Case report	2021	UK	Li et al. (46)
1M	82	1	Case report	2021	France	Launay et al. (47)
1F	41	1	Case report	2020	Peru	Añazco et al. (48)
1F	17	1	Case report	2020	UK	Zombori et al. (49)
4M	56.5	4	Case series	2020	Pakistan	Kazmi et al. (50)

Stages of AKI in patients with COVID-19 are presented in Table 2.

**Table 2:** Stage of AKI in patients with COVID-19 base on KDIGO Clinical Practice Guideline definition.

	**Stages**	**Number of**	**Number of patients**	***n*/*N* (%)**
		**studies**	**with COVID-19 and AKI**	
Stage of AKI	S1	2	3	3/3 (100%)
	S2	3	3	3/4 (75)
	S3	8	17	17/19 (89.5)

As shown in Table 3, AKI in admission was reported in 35 of 64 studied patients (56.2%), while 38 out of 51 patients (75.5%) showed AKI after admission.

**Table 3:** Summary of the case report and case series findings.

	**Variables**	**Number**	**Number of**	***n*/*N* (%)**
		**of**	**patients**	
		**studies**	**with**
			**COVID-19**
			**and AKI**
Demographic	AKI in admission	24	35	35/64 (56.2)
data	AKI after admission	19	38	38/51 (74.5)
	History of diabetes	23	40	40/100 (40)
	History of hypertension	29	59	59/96 (61.4)
	BMI (18.5–24.9)	8	31	31/44 (70.4)
	BMI: 25–29.9	11	20	20/34 (58.8)
	BMI ≥ 30	10	13	13/31 (41.9)
	BMI> 35	8	4	4/16 (25)
	IHD	9	7	7/27 (25.9)
	History of hyperlipidemia	7	12	12/21 (57.1)
	CKD history	12	12	12/54 (22.2)
Sign and symptoms	Dyspnea	22	33	33/39 (84.6)
	Shortness of breath	12	19	19/25 (76)
	Sore throat	5	5	5/5 (100)
	Cough	24	33	33/50 (66)
	Fever	30	41	41/45 (91.5)
	Malaria	11	12	12/15 (80)
	Fatigue	7	11	11/16 (68.7)
	Myalgia	15	16	16/29 (55.1)
	Shivering	3	4	4/7 (57.1)
	Abdominal pain	7	8	8/20 (40)
	Nausea and vomiting	10	12	12/30 (40)
	Diarrhea	7	7	7/15 (46.6)
	Loss of taste	3	3	3/7 (42.8)
	Loss of smell	1	1	1/2 (50)
	Edema	4	5	5/8 (62.5)
	Hypertension	13	24	24/34 (70.5)
	Hypotension	4	5	5/9 (55.5)
	Tachycardia	5	12	12/18 (66.6)
	Tachypnea	12	18	18/24 (75)
	SO2 > 93	10	11	11/13 (84.6)
	SO2 <93	20	34	34/37 (91.8)
Imaging	Lung involvement <50%	11	16	16/17 (94.1)
	Lung involvement >50%	9	10	10/14 (71.4)
	Consolidation	10	13	13/18 (72.2)
	PTE	1	1	1/3 (33.3)
	Sono, DVT	2	2	2/4 (50)
Pathology	FSGS	5	9	9/9 (100)
	AIN	3	6	6/7 (85.7)
	ATN	6	30	30/32 (93.7)
Prognosis	ICU admission	15	46	46/53 (86.7)
	Ventilation	16	49	49/56 (87.5)
	Septic shock	6	9	9/15 (60)
	Cardiogenic shock	1	5	5/8 (62.5)
	ARDS	14	17	17/36 (47.2)
	Death	14	49	49/74 (66.2)
	Recovered	28	50	50/96 (52.1)

*n, number of patients with any variables; N, the total number of studied patients; AKI, Acute kidney injury; IHD, Ischemic heart disease; HLP, Hyperlipidemia; CKD, chronic kidney disease; SO_2_, saturation oxygen; PTE, Pulmonary thromboembolic; DVT, Deep vein thrombosis; FSGS, focal segmental glomerulosclerosis; ATN, Acute tubular necrosis; AIN, Anal intraepithelial neoplasia; ICU, Intensive care unit; ARDS, Acute respiratory distress syndrome*.

BMI of patients with COVID-19 suffering from AKI was studied in 21 publications. The results showed that most patients (31/44) had a BMI in the range of 18.5–24.9 (Table 3).

The most common comorbidities were the history of diabetes, hypertension, and hyperlipidemia which were present in 40/100 (40%), 59/96 (61.4%), and 12/21 (57.1%) of patients with COVID-19 and AKI, respectively. According to the results of the included studies, 64.7% of patients received angiotensin-converting inhibitor/angiotensin receptor blocker (ACE/ARB), and 62.5% received diuretics. Also, 93.3% of the studied patients were taking oral diabetes medications. A history of chronic kidney disease (CKD) were reported in 12 out of the 54 evaluated patients (22.2%). More information about reported comorbidities in patients with COVID-19 and AKI can be found in Table 3.

Fever (91.5%), dyspnea (84.6%), shortness of breath (76%), cough (66%), hypotension (55.5%), loss of smell and taste (50%), and diarrhea (46.6%) were the most common symptoms. Based on the results of the included studies, 34 out of 37 patients (91.8%) showed a blood oxygen saturation level of less than 93%. However, ten other studies showed that 84.6% of patients had oxygen saturation levels higher than 93% (Table 3).

Imaging results from 9 studies demonstrated lung involvement in more than 50% of evaluated patients (71.4%). Computed tomography (CT) scans revealed that consolidation was another common finding in 72.2% of patients (Table 3).

Focal segmental glomerulosclerosis (FSGS) and acute tubular necrosis (ATN) were the most common pathological evidence (Table 3).

In terms of prognosis, intensive care unit (ICU) hospitalization and the need for a ventilator were reported in most of the involved patients (Table 3).

The average length of hospital stay was 19 days, and the average duration of need for mechanical ventilation was 3 days.

Most studies reported significant laboratory findings in patients with COVID-19 and AKI. 7/9 (77.7%), 4/4 (100%), and 13/18 (72.2%) of patients showed leukopenia, leukocytosis, and lymphopenia, respectively. High C-reactive protein (CRP) and low albumin were reported in 38/41 (92.7%) and 6/6 (100) of COVID-19 patients with AKI, respectively. Fifteen studies reported proteinuria, according to which 97.4% of patients (38/39) had this problem. Hematuria was seen in 21/34 (61.8%) patients from 11 studies. High creatine phosphokinase (CPK) was also reported in 8 studies (Table 4).

**Table 4:** Laboratory findings in the included studies.

	**Variables**	**Number**	**Number of**	***n*/*N* (%)**
		**of**	**patients**	
		**studies**	**with**
			**COVID-19**
			**and AKI**
Laboratory	Leukopenia	7	7	7/9 (77.7)
findings	Leukocytosis	4	4	4/4 (100)
	Lymphopenia	13	13	13/18 (72.2)
	Neutrophilia	3	3	3/4 (75)
	Thrombocytosis	6	8	8/9 (88.8)
	Normal platelet	9	12	12/14 (85.7)
	Anemia	9	9	9/22 (40.9)
	Normal hemoglobin	8	11	11/24 (45.8)
	High PTT	6	6	6/10 (60)
	High BUN	17	41	41/45 (91.1)
	High Urea	12	26	26/30 (86.7)
	High troponin	7	14	14/16 (87.5)
	Low albumin	4	6	6/6 (100)
	High CRP	22	38	38/41 (92.7)
	Abnormal ESR	7	16	16/17 (94.1)
	High lactate	9	20	20/24 (83.3)
	High IL-6	3	4	4/5 (80)
	d-dimer <1,000	8	8	8/10 (80)
	d-dimer:1,000–5000	8	9	9/12 (75)
	d-dimer:>5,000	4	8	8/9 (88.9)
	High LDH	13	15	15/23 (65.2)
	High ferritin	12	24	24/27 (88.9)
	High FBS	5	16	16/16 (100)
	High CPK	8	10	10/10 (100)
	Leukocyturia	4	7	7/18 (38.8)
	Oliguria	14	26	26/42 (61.9)
	Acidosis	9	19	19/19 (100)
	Proteinuria	15	38	38/39 (97.4)
	Hematuria	11	21	21/34 (61.8)
	Anuria	9	10	10/12 (83.3)
	Hypercalcemia	9	9	9/15 (60)
	Hypocalcemia	4	4	4/4 (100)
	Hyponatremia	8	8	8/9 (88.9)

*n, number of patients with any variables; N, the total number of studied patients; PTT, Partial thromboplastin time; BUN, Blood urea nitrogen; CRP, C-reactive protein; ESR, Erythrocyte sedimentation rate; IL, Interleukin; LDH, Lactate dehydrogenase; FBS, Fasting blood sugar; CPK, Creatine phosphokinase*.

As shown in Table 5, remdesivir, was the most frequently used antiviral agent. Intubation was also reported as the most common non-pharmacological treatment strategy. Furthermore, 33 out of 38 evaluated patients (86.8%) required hemodialysis.

**Table 5:** Treatment agents used in the included studies.

	**Variables**	**Number**	**Number of**	***n*/*N* (%)**
		**of**	**patients**	
		**studies**	**with**
			**COVID-19**
			**and AKI**
Treatment	O2 nasal	13	14	14/19 (73.7)
	O2 mask	4	5	5/8 (62.5)
	Venturi	4	4	4/4 (57.1)
	Intubation	24	70	70/78 (89.7)
	Hemoperfusion	3	3	3/3 (100)
	Hemodialysis	16	33	33/38 (86.8)
	CRRT	9	28	28/41 (68.3)
	Hydroxychloroquine	16	49	49/59 (83.1)
	Antibiotics	22	39	39/56 (69.6)
	Azithromycin	10	20	20/33 (60.6)
	Remdesivir	2	3	3/4 (75)
	Ritonavir	3	4	4/6 (66.7)
	Lopinavir	3	3	3/6 (50)
	Serum antibody	2	5	5/11 (45.4)
	Statins	2	4	4/5 (80)
	Heparin	8	11	11/11 (100)
	LMWH	10	37	37/39 (94.9)
	Tocilizumab	8	21	21/34 (61.8)
	A.S.A	5	20	20/28 (71.4)
	ACE/ARB	8	11	11/17 (64.7)
	β-Blocker	2	2	2/2 (100)
	CCB	4	6	6/9 (66.6)
	Steroids	2	2	2/3 (66.6)
	Oral Diabetes Medications	8	14	14/15 (93.3)
	Diuretic	4	5	5/8 (62.5)

*n, number of patients with any variables; N, the total number of studied patients; CRRT, Continuous renal Replacement therapy; ASA, Aspirin; ACEI/ARB, angiotensin converting inhibitor/angiotensin receptor blocker; CCB, Calcium channel blocker; LMWH, Low molecular heparin*.

## Discussion

In this systematic review, a total of 114 COVID-19 patients with AKI were identified. In prior studies, the prevalence of AKI in COVID-19 patients has been reported widely ranged from 0.5% in China by Guan et al. (51) to 80% in critically ill COVID-19 patients in France by Rubin et al. (52). Xu et al. reported that AKI incidence in COVID-19 patients was 10% and increased to 26% in the ICU-admitted subgroup of patients (1). Silver et al. demonstrated that AKI occurred in 30% of COVID-19 hospitalized patients and that the risk increased to more than 45% in patients requiring ICU admission. The heterogeneity in the reports of AKI incidence between studies could be explained by: (1) variation of the definition of “severe” disease, (2) the differences in admission criteria and hospital care, (3) genetic predisposition to kidney involvement, (4) differences in the frequency of kidney function measurement, and (5) kidney replacement therapy (KRT) resource limitations (53, 54).

In recent studies, kidney tissue sample analysis shed some light on potential pathophysiological mechanisms responsible for COVID-19 related AKI. Commonly cited hypotheses include: (1) tubular epithelial and podocyte damage due to highly expressed angiotensin-converting enzyme-2 (ACE2) in proximal tubular epithelial cells and podocytes, which serves as an entrance door for SARS-COVID-19, causing ATN (55–57), (2) direct infection of glomerular endothelia, causing FSGS (58), (3) COVID-19 related hypovolemia which leads to pre-renal AKI (59), (4) complement activation, cytokine storm, hypercoagulability and microangiopathy which can lead to multiple organ damage especially acute cardiac and lung injury and subsequent AKI via hypoxia and hypotension (55, 60, 61), (5) nephrotoxic drugs or contrast media (59), and (6) comorbidities like diabetes mellitus and hypertension which confer renal vulnerability to AKI (59).

As mentioned above, ATN and FSGS were the most common pathological findings in COVID-19 positive AKI in the current study. FSGS was pathologically investigated in all patients evaluated in the related studies (100%). Likewise, the pathological findings of ATN were also observed in 93.7% of patients.

The most common comorbidities reported in this systematic review, including the history of diabetes mellitus, hypertension, hyperlipidemia, and CKD, were present in 40%, 61.4%, 57.1%, and 22.2% of patients with COVID-19 and AKI, respectively. Based on the growing consensus and evidence, factors including older age, diabetes, hypertension, cardiovascular disease, high BMI, CKD, immunosuppression for any reason, and smoking are the potential risk factors for COVID-19 AKI (62–64). Some laboratory factors including leukocytosis, lymphopenia, elevated CRP, elevated ferritin (62), haematuria and proteinuria (65, 66) were also associated with COVID-19 AKI; which are reported 100, 72.2, 92.7, 88.9, 61.8 and 97.4%, respectively.

In terms of prognosis, ICU hospitalization and the need for assisted ventilation were commonly reported in 86.7 and 87.5% of involved patients, respectively. In patients with COVID-19 and AKI, the overall hospital mortality was 66.2%, comparable with early reports (67, 68).

AKI is considered an independent risk factor for increased mortality in critically ill patients of any disease, including COVID-19 (69). Kidney involvement has also been reported as an indicator of poor prognosis regardless of initial COVID-19 severity (68), which makes early detection and treatment of renal abnormalities improve the vital prognosis of COVID-19 patients.

According to the previous studies, the burden of CKD following COVID-19-related AKI may be substantial, and AKI has been linked to an increased risk of CKD in individuals with previously normal renal function (70, 71). It is essential to say that pre-existing CKD and AKI have been described as predictors of severe and critical illness in patients with COVID-19, with a higher mortality rate than patients without kidney deficiency.

The lack of effective treatments for patients with COVID-19 and AKI has required repurposing several drugs, including remdesivir. The current systematic review indicated that remdesivir, was the most frequently used antiviral agent. These compounds may induce AKI and are not recommended in patients with poor renal function. Thus, early detection and specific therapy of renal changes, including adequate hemodynamic support and avoidance of nephrotoxic drugs, may help to improve critically ill patients with COVID-19.

Our systematic review has some limitations. First, since included studies were case reports articles with a low number of patients, the generalizability of our findings may be limited. Second, detailed information on patient characteristics was lacking in the published articles, and the potential influence of pre-existing conditions could not be investigated. Furthermore, studies' variability and different patients' characteristics were other limitations.

In conclusion, this systematic review shows that AKI frequently complicates the course of COVID-19 hospitalizations and is associated with increased severity of illness, prolonged duration of hospitalization, and poor prognosis. Given the extent of the adverse impact of AKI, it is imperative that early detection of comorbidities and renal complications is essential to improve the outcomes of COVID-19 patients. Further research on large scales is warranted to improve our understanding of this disease and design clinical approaches to managing COVID-19 related AKI.

## Data Availability Statement

The original contributions presented in the study are included in the article/supplementary material, further inquiries can be directed to the corresponding author/s.

## Author Contributions

TS, MN, and MM designed the study. TS, AK, FO, SK, BH, RV-H, AT, AS, and SO performed the search, study selection, and data extraction. TS, AK, BH, MN, SH, and AA wrote the first draft of the manuscript. MN, BH, AS, and MM revised the article. All authors approved the submitted version.

## Funding

MN and his colleagues from Iran were financially supported by a grant from Shahid Beheshti University of Medical Sciences, Tehran, Iran.

## Conflict of Interest

The authors declare that the research was conducted in the absence of any commercial or financial relationships that could be construed as a potential conflict of interest.

## Publisher's Note

All claims expressed in this article are solely those of the authors and do not necessarily represent those of their affiliated organizations, or those of the publisher, the editors and the reviewers. Any product that may be evaluated in this article, or claim that may be made by its manufacturer, is not guaranteed or endorsed by the publisher.
